# Draft Genome Sequence of Truncatella angustata (Anamorph) S358

**DOI:** 10.1128/mra.00052-22

**Published:** 2022-06-06

**Authors:** Harald Kellner, Stephanie Friedrich, Kai-Uwe Schmidtke, René Ullrich, Jan Kiebist, Daniel Zänder, Martin Hofrichter, Katrin Scheibner

**Affiliations:** a Department of Bio- and Environmental Sciences, International Institute Zittau, Technische Universität Dresden, Zittau, Germany; b Institute of Biotechnology, Brandenburg University of Technology Cottbus-Senftenberg, Senftenberg, Germany; c JenaBios GmbH, Jena, Germany; University of California, Riverside

## Abstract

The ascomycete Truncatella angustata has a worldwide distribution. Commonly, it is associated with plants as an endophyte, pathogen, or saprotroph. The genome assembly comprises 44.9 Mbp, a G+C content of 49.2%, and 12,353 predicted genes, among them 12 unspecific peroxygenases (EC 1.11.2.1).

## ANNOUNCEMENT

Truncatella angustata (Pers.) S. Hughes 1958 (1) belongs to the Sporocadaceae, a family of coelomycetous fungi with appendage-bearing conidia within the ascomycetous order Xylariales (2). It is common as an endophyte or pathogen of vascular plants in both temperate and tropical regions (3, 4). It infects stems (*Vitis* [5, 6], *Vaccinium* [7]), leaves (*Rosa* [8], *Parthenocissus* [3], *Populus* [9]), fruits (*Malus* [10], *Olea* [11]), and roots (*Vitis* [12]) and is also a candidate for biological control of plant diseases (9, 12). In addition to plants, this fungus was also isolated from marine sponges (13), humans (4), and as a pathogen from insects (14). *T. angustata* cultures showed several secondary metabolites with potential for application in biotechnology or medicine, e.g., α-pyrone-based analogs (15), phenazine-1-carboxylic acid with antifungal activity (6), ramulosin derivates with a broad range of biological activities (14, 16), and truncateols, isoprenylated cyclohexanols with antiviral activity (15, 17). In culture supernatants, isolate S358 showed activities of several oxidoreductases, including those of unspecific peroxygenase and laccase. Its genome will be useful for identifying biotechnologically relevant enzymes or biosynthetic clusters.

*T. angustata* isolate S358 (rRNA genes and internal transcribed spacer [ITS], GenBank accession number OL604502) was collected from a fruiting body of the basidiomycetous species Psathyrella conopilus, which was growing on soil mulched with Robinia pseudoacacia wood chips (Bernsdorf, Germany; 51°23′51.1″N, 14°01′42.2″E). The fungus was cultured at 24°C and 120 rpm for 3 days in a synthetic medium (18) inoculated with a conidiospore suspension. Mycelium was harvested by vacuum filtration, washed twice, and lyophilized. Genomic DNA was extracted using the FastDNA Spin kit for soil (MP Biomedicals, Germany) from 30 mg of the harvested material. Sequencing libraries were prepared using the NEBNext Ultra II DNA library prep kit (New England Biolabs, Frankfurt, Germany), and genome sequencing was performed using an Illumina NextSeq 500 instrument in 2 × 150-bp paired-end read mode. After quality and adapter filtering using BBDuk v38.84, a total of 26 million reads were used for *de novo* assembly using SPAdes v3.15.2 (19) with default parameters. The assembly consists of 853 contigs with a total length of 44.9 Mbp. The assembly was verified using QUAST v5.0.2 (20) and has an *N*_50_ value of 102,256 bp and a G+C content of 49.2%; the largest contig has a size of 376,640 bp. The completeness of the assembly was verified using BUSCO v5 (data set, ascomycota_odb10) and determined to be 93.8% (21). Gene prediction was performed using AUGUSTUS v3.4 (22) (predictor: Fusarium graminearum, both strands, only complete genes, without in-frame stop codons) and resulted in 12,353 protein-coding genes. The genes were annotated using OmicsBox v2.0.36 (23) (BioBam, Valencia, Spain) following a pipeline of blastp-fast search (E value, 1.0E-3; word size, 6), InterProScan (all member databases), and GO mapping (Goa v2021.11). Carbohydrate-active enzymes (CAZymes) were identified using dbCAN2 (HMMdb v10; E value, <1e-15; coverage, >0.35) (24). Altogether, 366 glycoside hydrolases, 65 carbohydrate esterases, 29 polysaccharide lyases, 102 glycosyltransferases, 183 enzymes with auxiliary activities, and 15 carbohydrate-binding modules (CBM) were identified (Table 1).

**TABLE 1:** CAZyme classes, unspecific peroxygenases, and multicopper oxidases detected in the genome of *Truncatella angustata* S358

Enzyme or domain group	No. of proteins	GenPept accession no.
Glycoside hydrolases	366	
Glycosyl transferases	102	
Polysaccharide lyases	29	
Carbohydrate esterases	65	
Enzymes with auxiliary activity	183	
Associated modules		
Carbohydrate-binding modules	15	
Cellulose-binding domain CBM1	0	
Enzymes of interest		
Multicopper oxidase	19	KAH8193788, KAH8193910, KAH8196607, KAH8197187, KAH8197370, KAH8199586, KAH8199725, KAH8199806, KAH8200567, KAH8200848, KAH8201626, KAH8201963, KAH8202890, KAH8203692, KAH8204301, KAH8204821, KAH8204968, KAH8205436, KAH8205682
Unspecific peroxygenase	12	KAH8195642, KAH8196030, KAH8197040, KAH8199887, KAH8200077, KAH8200361, KAH8201371, KAH8202439, KAH8203164, KAH8203310, KAH8203546, KAH8204654

Using the unspecific peroxygenase (UPO; EC 1.11.2.1) reference sequence from Cyclocybe (Agrocybe) aegerita (GenPept accession number CBJ94532), 12 putative UPO genes were detected in *T. angustata*. Further, 19 multicopper oxidases were identified, among them 12 laccases. Moreover, 82 secondary metabolite biosynthetic gene clusters (BGCs) were predicted using antiSMASH v6 (25) (using contigs; detection strictness, relaxed), among them 40 related to the synthesis of polyketides, 35 to nonribosomal peptides, and 11 to terpenes.

### Data availability.

This whole-genome shotgun project has been deposited at DDBJ/ENA/GenBank under the accession number JAJJMK000000000. The version described in this paper is version JAJJMK010000000. The Sequence Read Archive (SRA) accession number is SRR16694223.
